# Urolithin A (UA) attenuates ferroptosis in LPS-induced acute lung injury in mice by upregulating Keap1-Nrf2/HO-1 signaling pathway

**DOI:** 10.3389/fphar.2023.1067402

**Published:** 2023-03-09

**Authors:** Lejing Lou, Min Wang, Jingjing He, Song Yang, Fanxi Meng, Shijia Wang, Xiao Jin, Jihao Cai, Chang Cai

**Affiliations:** ^1^ Department of Respiratory and Critical Care Medicine, The First Affiliated Hospital, Wenzhou Medical University, Wenzhou, China; ^2^ School of Pharmaceutical Science, Wenzhou Medical University, Wenzhou, China; ^3^ Renji College of Wenzhou Medical University, Wenzhou, China

**Keywords:** urolithin a, Nrf2, ferroptosis, acute lung injury, LPS

## Abstract

Acute lung injury (ALI) is a life-threatening disease with high incidence and mortality rates. Urolithin A (UA) is a pomegranate intestinal flora metabolite with anti-inflammatory, antioxidant, and anti-aging properties. Ferroptosis is a critical factor in lipopolysaccharide (LPS)-induced acute lung injury (ALI). However, the link between UA and ferroptosis is unknown. The purpose of this research was to look into the role of UA in regulating LPS-induced ferroptosis in ALI. The current study used LPS to injure two models, one BEAS-2B cell injury model and one ALI mouse model. UA effectively alleviated LPS-induced ALI compared to the LPS group by lowering *in vivo* lung wet/dry weight ratio, reactive oxygen species, and malondialdehyde production, as well as superoxide dismutase, catalase, and glutathione depletion. Furthermore, by increasing GPX4 and SLC7A11 expression and decreasing Fe^2+^ levels, lung histopathological damage, inflammatory cytokine secretion, and ferroptosis levels can be significantly reduced. The Keap1-Nrf2/HO-1 pathway was upregulated by UA, which inhibited LPS-induced ALI and ferroptosis. ML385 inhibited UA’s protective effect against LPS-induced ALI. These findings suggested that UA could be a novel potential therapeutic target for ALI.

## Introduction

The severe respiratory disorder known as acute lung injury (ALI) is characterized by uncontrollable pulmonary edema, oxidative stress (OS), and infiltration of inflammatory cells and has a high mortality rate (Fan et al., 2018). The pathophysiology and pathogenesis of ALI are still not fully elucidated. As ALI is aggravated, acute respiratory distress syndrome (ARDS) may develop (Ware and Matthay, 2000). Currently, there is no effective treatment for ALI that can significantly raise life expectancy and reduce mortality (Beitler et al., 2022; Yaqub et al., 2022). Therefore, uncovering additional information about the pathophysiological and pathogenic mechanisms of ALI and developing efficient therapeutic agents are of great significance.

The type of programmed cell death that is iron-dependent and distinct from apoptosis, which exhibits the features of reactive oxygen species (ROS) accumulation, is called ferroptosis (Dixon et al., 2012). Ferroptosis differs from other types of cell death in terms of both mechanism and phenotype (Xie et al., 2016). Recently, ferroptosis has significantly impacted the frequency of ALI (Liu et al., 2020; Xu et al., 2020; Liu et al., 2021). The levels of ferroptosis markers GPX4 and SLC7A11 significantly decreased while free iron content within bronchial epithelial cells (BECs) in ALI mice increased in the lipopolysaccharide (LPS)-induced ALI mouse model (Liu et al., 2020). However, lung injury was clearly reduced in mice treated with ferroptosis inhibitor (ferrostatin-1), demonstrating the crucial function of ferroptosis in the development and progression of LPS-mediated ALI (Liu et al., 2020). The apoptosis of alveolar epithelial cells (AEC) plays a key role in the progression of ALI (Liu et al., 2022). According to Wang et al. (Wang et al., 2022), the AU-rich element (ARE)-binding factor 1 (AUF1) was found to alleviate cecum ligation and puncture (CLP) -induced ALI by inhibiting alveolar epithelial ferroptosis. Therefore, inhibition of ferroptosis of AECs might serve as the crucial anti-ALI therapeutic target. Ferroptosis has been confirmed as a therapeutic target for acute lung injury in many animal or cellular models of ALI, but the exact mechanisms have not been fully elucidated (Yin et al., 2021).

NRF2 is a crucial regulator of ferroptosis and lipid peroxidation that transcriptionally regulates diverse critical components in the anti-ferroptotic pathway (Dodson et al., 2019). One recent study found that Nrf2 regulates HO-1 and SLC7A11 to suppress ferroptosis, which improved the effect of gut ischemia-reperfusion (IR)-induced ALI (Dong et al., 2020). Qiu and colleagues reported that Nrf2 prevents ALI caused by seawater drowning by inhibiting ferroptosis (Dong et al., 2020). Li and colleagues also reported that panaxydol suppressed ferroptosis in LPS-mediated ALI *via* KEAP1-NRF2/HO-1 pathway (Li et al., 2021). According to Li and colleagues, obacunone promoted Nrf2-mediated antioxidation, reducing ferroptosis in the LPS-mediated ALI (Li et al., 2022). The novel strategy to treat ALI is possible because ferroptosis promotes ALI development while activating Nrf2 can suppress ferroptosis. Therefore, it is essential to investigate the role of Nrf2 activation in ALI-induced ferroptosis and develop inhibitory therapeutic agents.

One of the natural metabolites of ellagitannins and ellagic acid (Cerda et al., 2005), which are abundant in pomegranates, strawberries, and other nuts, is Urolithin A (UA), which is derived from the gut microbiome (D'Amico et al., 2021). Prior research has revealed that UA has different pharmacological properties, including antioxidation (Casedas et al., 2020), anti-inflammation (Larrosa et al., 2010; Singh et al., 2019), neuroprotection (Y. L. Chen et al., 2021), and improved muscle function (Luan et al., 2021). However, it has been unclear what role UA plays in acute lung injury. Recent studies have shown that UA can reduce acetaminophen-mediated liver necrosis and OS by the activation of the Nrf2/ARE pathway (Gao et al., 2022). Through the p62-Keap1-Nrf2 pathway, UA reduced acute kidney injury (AKI) resulting from renal ischemia reperfusion (Zhang et al., 2022). Therefore, it has been hypothesized that UA can ameliorate LPS-mediated ALI by promoting Nrf2-mediated antioxidation.

In this study, we found that UA effectively reduced OS and inflammatory injury caused by ALI. Additionally, UA suppressed ferroptosis and ROS generation caused by LPS *in vitro* and *in vivo*. Our findings showed that UA activated Nrf2 to suppress LPS-mediated inflammation and ferroptosis. Our study also demonstrated that UA is effective in treating ALI and explored the mechanism of inhibiting the effect of ALI-induced ferroptosis.

## Materials and methods

### Reagents and antibodies

Urolithin A (UA, ≥97% purity) was provided by Sigma-Aldrich (St Louis, MO, United States). ML385 and Erastin were provided by MedChem Express (Monmouth Junction, NJ, United States). Lipopolysaccharides (LPS) were provided by Sigma-Aldrich (St Louis, MO, United States). The anti-Nrf2, anti-GPX4, anti-HO-1, anti-SLC7A11, and anti-NQO1 primary antibodies were provided by Proteintech Group (Wuhan, China). Lamin B and *β*-actin antibodies were provided by Cell Signaling Technologies (Danvers, MA, United States). 4-HNE used in immunohistochemical (IHC) staining was provided by Abcam (Cambridge, United Kingdom). Reagents for cell culture were provided by Gibco (Grand Island, NY). The Alexa Fluor^®^488-labeled Goat Anti-Rabbit IgG secondary antibody (H + L), 4′, 6-diamidino-2-phenylindole (DAPI), and cell live/dead staining kits were provided by Yeasen Bio. Inc., (Shanghai, China).

### Cell culture and treatment

A human bronchial epithelial cell line BEAS-2B (Procell, Wuhan, China) was cultured in RPMI-1640 containing 1% antibiotics and 10% FBS at 37°C and 5% CO_2_. The media was changed every 2-day. At ∼ 80%–90% confluence, cells were passaged. In addition, LPS (10ug/mL) treatment of BEAS-2B cells can effectively cause cell damage (J. Li et al., 2022).

### Cell viability test

BEAS-2B cells (5 × 10^3^/well) were seeded in 96-well plates and incubated in a 100 µL medium overnight to detect the cytotoxic effect of UA. After that, UA at different concentrations (0, 2.5, 5, 7.5, 1 0, 20, and 40 µM) were used to treat the medium. CCK-8 reagent (10 µL) was added to all the wells after 24/48 h, followed by 1.5 h incubation in the dark. The microplate reader was used to measure the absorbance (OD) at 450 nm.

### Animal’s model

This study obtained the 8–10-week-old wild-type (WT) male C57BL/6 mice (20–24 g) from the Animal Center of the Chinese Academy of Sciences Shanghai, raised them under SPF-condition, and used them after 1 week of acclimatization. The Animal Ethics Community of the First Affiliated Hospital of Wenzhou Medical University approved each animal procedure. The mice were randomly divided into five groups: Control, LPS, LPS + UA, LPS + UA + Erastin, and LPS + UA + ML385 groups. Dimethyl sulfoxide (DMSO) was used to dissolve Urolithin A and ML385. ML385 was injected intraperitoneally 30 min before the LPS challenge. Mice were anesthetized and intratracheally injected with 10 mg/kg LPS to induce the LPS-induced ALI model; the same volume of PBS was injected into control mice, while mice of the LPS + UA group were exposed to intraperitoneal injection of 50 mg/kg UA after LPS (10 mg/kg) challenge. The ferroptosis agonist Erastin (15 mg/kg) was mixed with 5% dimethyl sulfoxide (DMSO) and administrated intraperitoneal to mice 1 h before UA administration. Before LPS treatment, 30 mg/kg ML385 was administered to the LPS + UA + ML385 group, followed by UA. Afterward, each mouse was placed in a perpendicular position, followed by slow shaking for a 1-min period to ensure the even distribution of LPS or PBS between bilateral lungs. Each mouse was sacrificed after 24 h of LPS treatment. Subsequently, lung tissues and bronchoalveolar lavage fluid (BALF) were collected.

### Western-blot (WB) assay

Cells were lysed in RIPA buffer containing 1% PMSF, and total cellular protein was extracted. In brief, cells were lysed with lysate for a 15-min on ice, followed by 30-min ultracentrifugation at 12,000 rpm at 4 C to obtain cellular protein. The protein content was then detected using the BCA assay kit (Beyotime, Shanghai, China). Later, proteins (40 ng) were separated using AGE, followed by transfer onto the PVDF membrane (Bio-Rad, California, United States). After that, the membrane was blocked for a 1-h with 5% defatted milk, rinsed thrice with TBST (Tris-buffered saline containing Tween-20), cut, and incubated with different primary antibodies overnight at 4°C. After three washes with TBST. The membranes were incubated with a secondary antibody for 1 h at room temperature. Finally, membranes were rinsed three times with TBST before using Chemi DocXRS + imaging system (Bio-Rad) to detect protein blots using the super-sensitive ECL chemiluminescence kit. For quantification, ImageJ software was used.

### BALF cell counting, protein concentration, and pro-inflammatory cytokines

The trachea was exposed after the thoracic cavity and neck were opened after the mice were executed. Tracheal intubation was performed, and 1 mL PBS was instilled into the lungs before being aspirated three times with a catheter-connected syringe. BALF was centrifuged for 4 min at 3,000°C at 10 rpm, and the cell precipitate and supernatant were collected separately. After resuspending the cell precipitate in PBS, the total cells were counted using a hematocrit counter. The BCA protein assay kit (Beyotime, Shanghai, China) was used to measure protein contents in BALF according to the manufacturer’s instructions. The pro-inflammatory factors TNF-*α*, IL-1β, and IL-6 were also measured using ELISA kits (Beyotime, Shanghai, China).

### Lung wet/dry (W/D) ratio

The whole lung tissue was dissected and washed with PBS solution; after that lung surface was dried using absorbent paper, followed by immediate weighing to obtain wet weight. Afterward, samples were baked in an oven at 55°C for a 48-h before being weighed to obtain a dry weight. Later, the W/D ratio was determined to assess the severity of tissue edema.

### Histopathological evaluation

The Left lung of mice was removed and fixed in 10% neutral formalin for 48 h. Dehydration of lung tissues was accomplished using different concentrations of alcohol, followed by paraffin embedding and sectioning to the 4-µm sections. Thereafter, hematoxylin and eosin (H&E) staining was performed on the obtained sections, and pathological analysis was done using an optical microscope.

### Detection of intracellular ROS generation and mitochondrial ROS production

Intracellular were measured using the Reactive Oxygen Species Assay Kits as directed by the manufacturer (Beyotime, Shanghai, China). Briefly, cells were incubated with fresh DMEM medium containing 10 μM DCFH-DA at 37°C for 30 min. Then the cells were washed twice with PBS and resuspended in cold PBS for flow cytometry analysis. Mitochondrial ROS levels in appropriately treated BEAS-2B cells were determined by staining with MitoSox according to the manufacturer’s instructions (Thermo Fisher Scientific). A fluorescence microscope was used to examine stained cells (Olympus Life Science; Tokyo, Japan).

### Quantification of MDA, GSH-Px, SOD, and CAT activities

Following the sacrifice of the mouse, the right lung tissue was dissected. Later, extraction buffer was added to the lung tissue homogenates. Finally, the levels of SOD, MDA, CAT, and GSH-Px were determined using commercial assay kits (Jiancheng Biotechnology, Nanjing, China) as directed by the manufacturer.

### Fe^2+^ detection

Cell lysate (200 µL/well) was rinsed with PBS twice and then put on shaking for 2 h. After that, standard and mix A dilutions were completed per specific protocols (Abcam (Cambridge, United Kingdom). Samples from different groups were combined and incubated at 60°C for 1 h. The sample was cooled and centrifuged. 30 μL of iron ion detection agent was added to the samples, and all the samples were incubated for 30 min at room temperature. Thereafter, each well of the 96 well-plate was added with solution (200 µL), and the OD value was measured at 550 nm. Finally, iron ion content was determined, and a standard curve was drawn.

### Immunofluorescence (IF) staining

Cell sections were rinsed with PBS (three times)and fixed for 30-min using 4% paraformaldehyde (PFA), followed by another 3-min treatment using 0.3% Triton X-100. Then sections were washed with PBS (three times), and 10% BSA was added to seal cells for a 30-min, followed by overnight incubation with primary antibodies (1:200) at 4°C. Next day, fluorescent secondary antibodies (1:300) were added to cells and incubated in the dark for a 60-min at room temperature, followed by 8-min DAPI (1:4,000) staining before fluorescence microscopy (Olympus Life Science; Tokyo, Japan). All codomain computations were performed in five separate experiments, each with 50 cells. The images were demonstrated using Adobe Photoshop 6.0. Individuals blinded to the treatments determined fluorescence intensities using Image J software (Bethesda, United States).

### Immunohistochemical (IHC) staining

Lung tissues were fixed in 4% PFA, embedded in paraffin, and cut into 4-µm sections. The sections were dewaxed with xylene before being rehydrated with gradient ethanol. Sections were treated with 3% hydrogen peroxide to block endogenous peroxidase activity, and non-specific binding was inactivated with 10% goat serum. Then, sections were incubated overnight with anti-4-HNE, anti-GPX4 and anti- Nrf2 antibodies at 4°C, followed by 1-h incubation with HRP-labeled secondary antibodies at 37 °C. By adding a fresh DAB reaction mixture, color rendering was observed. The sections were dehydrated and sealed after being counterstained with hematoxylin. Finally, each section was photographed using a light microscope. The blinded observers quantified the rate of positive cells in each section. Five mice from each group were employed for the quantitative assessment.

### Transmission electron microscopy (TEM)

To fix lung tissues, 2.5% glutaraldehyde with 0.05 M sodium cacodylate buffer (pH 7.2) was added and incubated at 25°C for a 2-h. Additionally, 2% OsO4 with 0.1 M sodium cacodylate buffer was added for 2-h fixation, and 1% aqueous uranyl acetate was added for 18-h fixation. Following ethanol dehydration, samples were embedded in Epon 812 and collected in ultrathin sections onto the copper grids. Later, Lead citrate and uranyl acetate were added to examine the sections with a Tecnai G2 spirit BioTwin transmission electron microscope (FEI Company, Hillsboro, Oregon).

### Molecular docking (MD)

For MD analysis to determine the Nrf2/UA start structure for subsequent treatment, Discovery Studio 3.1 was used. The crystal structures of the Nrf2/Keap1 complex were obtained from the Protein Data Bank database (PDB ID: 1X2R). The minimum energy conformations for MD were established using default parameters, and PyMoL (version 1.7.6) was adopted for treatment. The molecular structures of Nrf2-Keap1 and UA’s were generated for MD analysis using AutoDockTools (version 1.5.6). PyMoL was used to view the final images in 3D.

### Statistical analysis

The statistical analysis was done using GraphPad Prism 8 software. Results were represented by mean ± SD. The Kruskal-Wallis test was used to determine lung injury scores across several groups. Multiple groups were compared using One-way ANOVA, and the statistical significance level was *p* < 0.05.

## Results

### Cytotoxic effect of UA on BEAS-2B Cells

**FIGURE 1:**
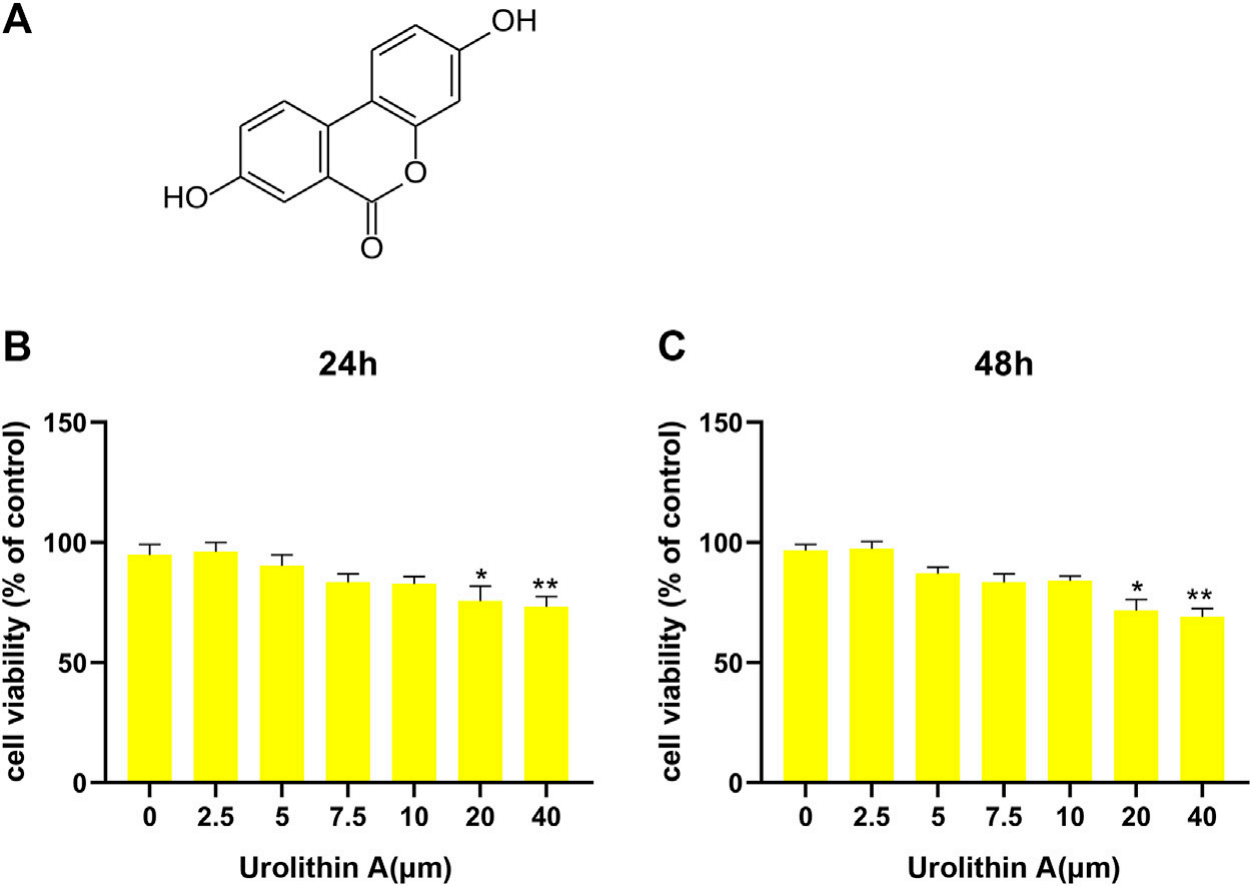
Effects of UA treatment on the viability of BEAS-2B cell. **(A)** The molecular formula of Urolithin A (UA). **(B,C)** CCK-8 results of BEAS-2B cells after 24/48 h UA treatments at diverse doses. Results were Shown as mean ± SD. *p < 0.05, **p < 0.01 in comparison to the control group.

UA had the molecular formula of C13H8O4, as shown in Figure 1A. For assessing UA’s cytotoxic effect on BEAS-2B cells, UA (2.5, 5, 7.5, 10, 20, 40 µM) was added to treat cells for 24/48 h. Then cell viability was measured using CCK-8 assay. Consequently, 24/48-h of 20 µM UA treatment reduced cell viability, whereas no cytotoxic effect of UA was seen at the dose of 0–10 µM (Figures 1B, C). Thus 10 µM was selected to be used as content in later experiments.

### Treatment with UA inhibited inflammatory responses and alleviated LPS-mediated injuries in BEAS-2B Cells and ALI mice

**FIGURE 2:**
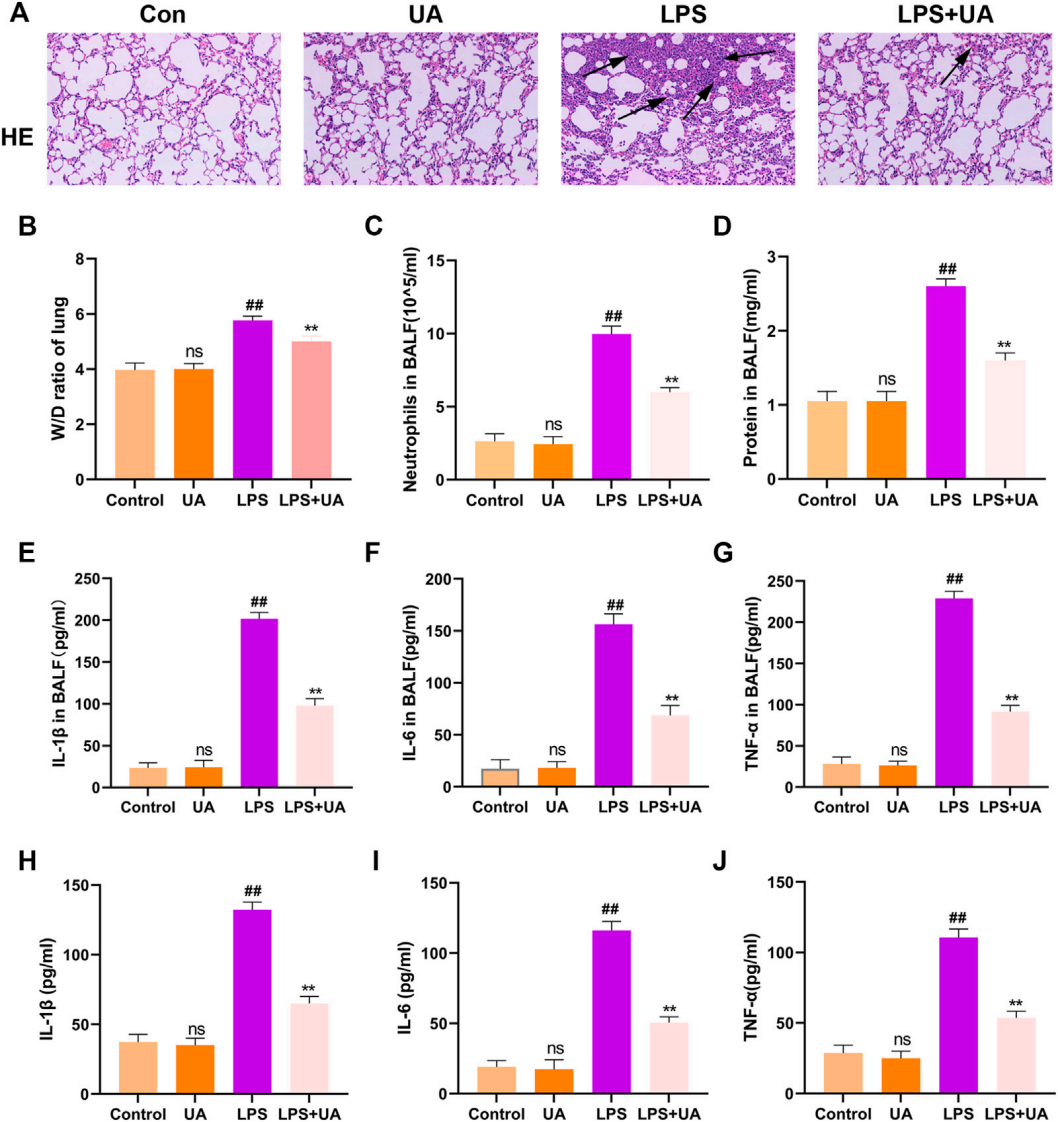
Role of Urolithin A (UA) in LPS-mediated ALI in BEAS-2B cells and mice. **(A)** H&E staining images of Lung tissue. **(B)** Lung W/D weight ratio. **(C,D)** Neutrophil number and protein content of the BALF. **(E–G)** TNF-*α*, IL-1β, and IL-6 expressions in mouse BALF were measured by ELISA assay. **(H–J)** TNF-*α*, IL-1β, and IL-6 expression in BEAS-2B cells culture supernatant treated with LPS(10 ug/mL) were analyzed through ELISA assay. The results were shown as mean ± SD. ##*p* < 0.01, **p < 0.01 in comparison to control and LPS groups, respectively.

H&E was added to stain mouse lung tissues for pathological analysis, and the effectiveness of UA in preventing LPS-induced ALI was analyzed. Compared to the control group, Mouse lung tissues from the LPS-mediated ALI model group showed distinct and specific pathological alterations, including decreased alveolar cavity, alveolar wall edema and congestion, and significant infiltration of inflammatory cells. Compared to the LPS-mediated ALI model group, the lung tissue structure of LPS + UA animals was nearly normal (Figure 2A). Lung W/D ratio and protein leakage in BALF were also used to determine edema severity, and LPS-mediated W/D ratio and protein content significantly increased as compared to the control group. In contrast, UA significantly reduced protein leakage and lung edema (Figures 2B, D).

In the mouse model, LPS treatment significantly increased neutrophil count compared to the control group, but UA treatment efficiently suppressed the LPS-mediated increase in neutrophil count (Figure 2C). Additionally, an ELISA assay was performed to measure the level of TNF-*α*, IL-1β, and IL-6 in BALF to analyze the effect of UA on LPS-mediated inflammatory responses. Our results suggested that UA effectively reduced the levels of TNF-*α*, IL-1β, and IL-6 (Figures 2E–G). According to the above *in-vivo* results, this study explored the role of UA treatment in inhibiting LPS-mediated inflammatory responses in BEAS-2B cells. In BEAS-2B cells, LPS treatment significantly increased the level of IL-1β, TNF-*α*, and IL-6, while UA significantly reduced LPS-induced IL-1β, IL-6, and TNF-α generation, as shown in Figures 2H–J.

### Treatment with UA reduced oxidative stress during LPS-mediated injuries to BEAS-2B Cells and ALI mice

**FIGURE 3:**
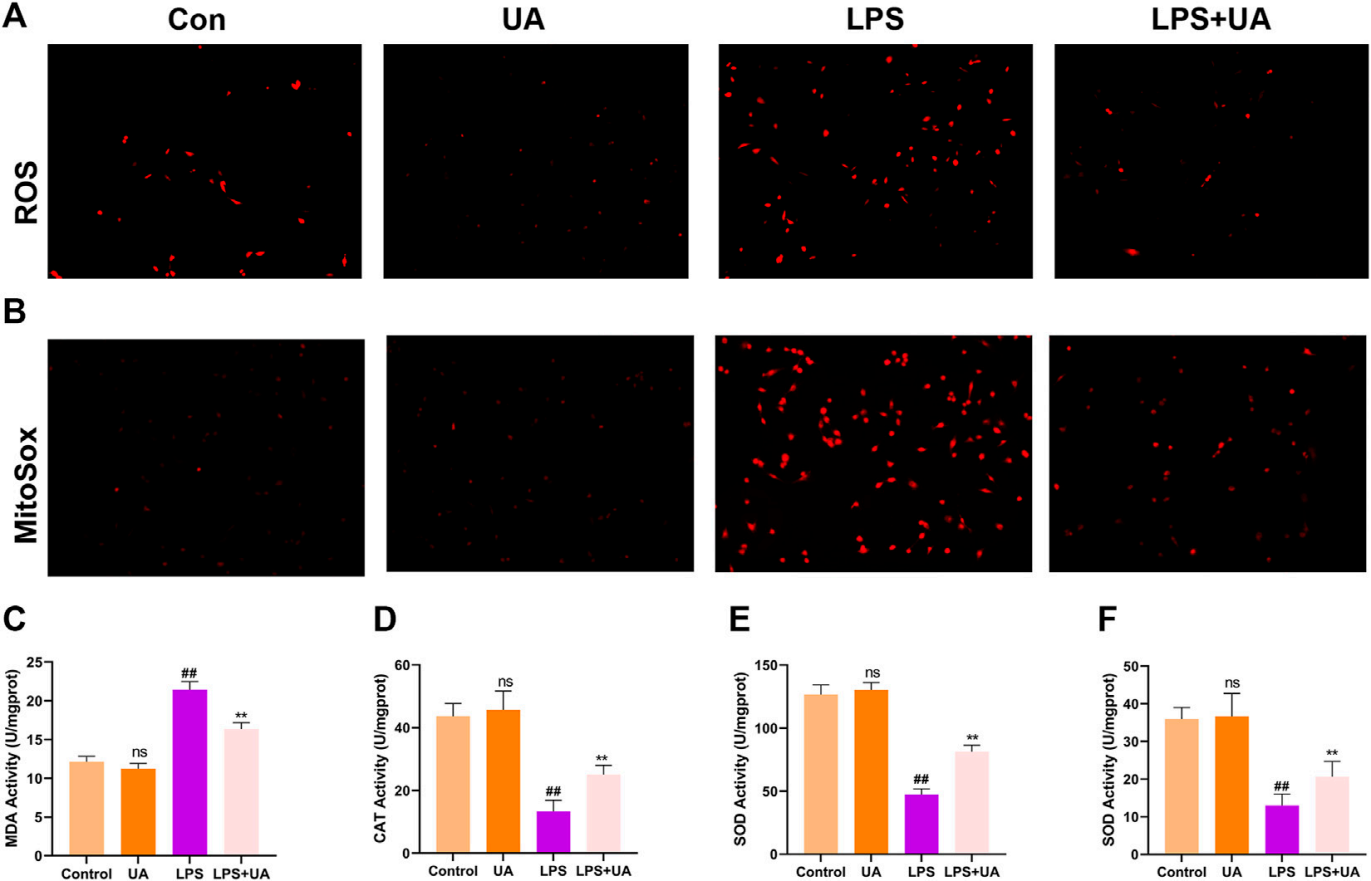
Effects of Urolithin A (UA) on LPS-mediated OS in ALI. **(A)** Typical images showed the ROS level in BEAS-2B cells. **(B)** Typical images showed MitoSox in BEAS-2B cells. **(C–F)** MDA, CAT, GSH, and SOD activities in the mouse lung tissues. All data were shown as mean ± SD. ##*p* < 0.01, **p < 0.01 in comparison to control and LPS groups, respectively.

This study analyzed the role of UA treatment in suppressing LPS-mediated OS *in vitro* and *in vivo* since Oxidative damage significantly promoted LPS-mediated ALI; We wanted to see if pretreatment with UA could prevent LPS-induced oxidative stress. Intracellular ROS and mitochondrial ROS can respond to a certain extent to the level of oxidative stress (L. Chen et al., 2020). The result shows that UA treatment significantly decreased LPS-induced ROS accumulation and mitochondrial ROS production in BEAS-2B cells (Figures 3A, B). MDA is a significant marker of OS, whereas SOD, CAT, and GSH-Px are significant markers of anti-OS. MDA contents in mice treated with UA significantly decreased compared to those in the LPS group (Figure 3C). Additionally, UA reversed CAT, SOD, and GSH-Px activities; however, after LPS treatment, these activities were decreased (Figures 3D–F). In BEAS-2B cells and ALI animals, UA thus decreased oxidative stress during LPS-mediated injury.

### UA suppressed ferroptosis and reduced LPS-mediated BEAS-2B injury and ALI

**FIGURE 4:**
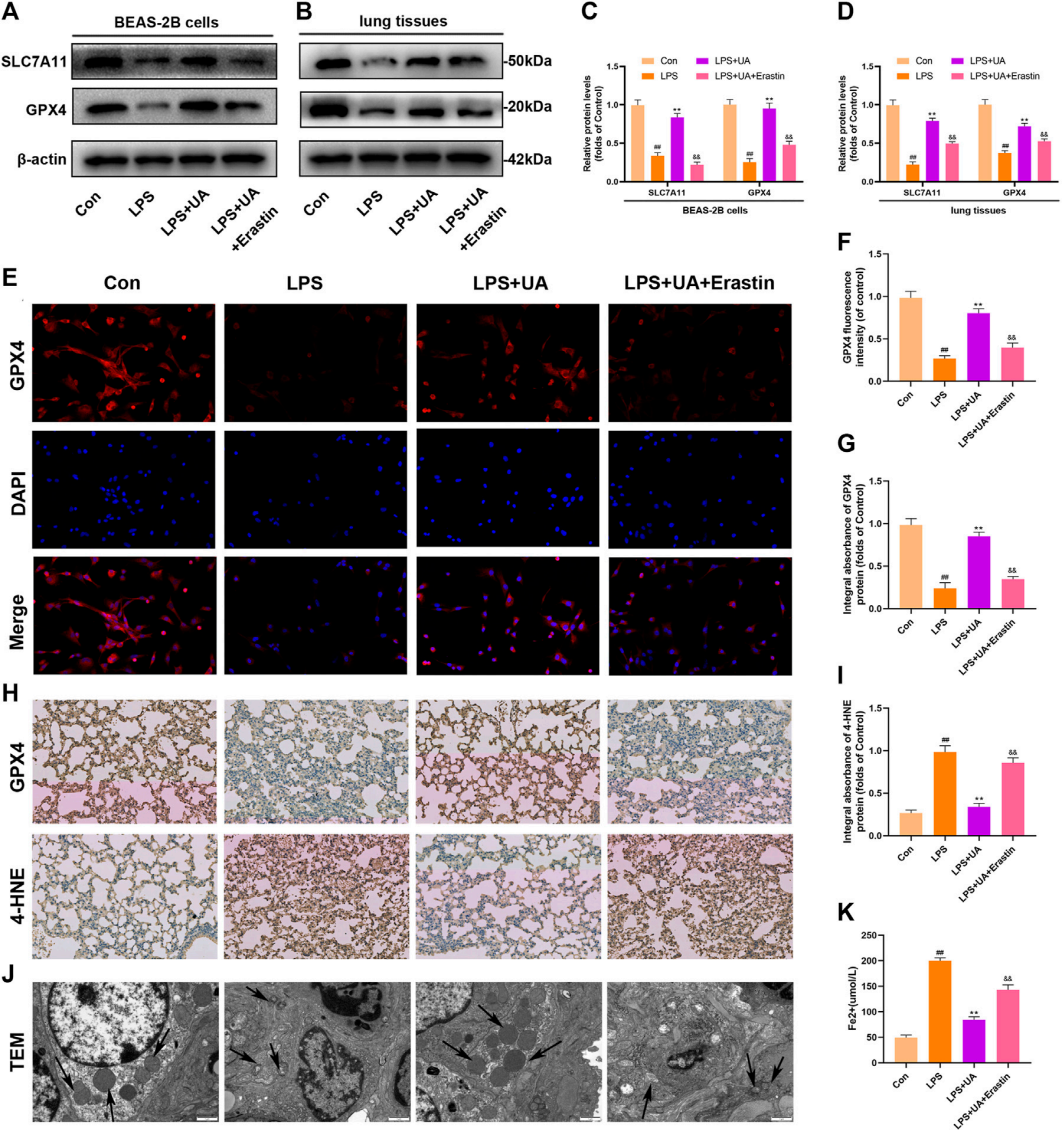
Role of UA in Ferroptosis during LPS-mediated injury *in vitro* and *in vivo*. **(A,C)** Levels of SLC7A11 and GPX4 in BEAS-2B cells were measured. **(B,D)** Quantification of SLC7A11 and GPX4 levels in lung tissues. **(E,F)** Representative immunofluorescence images of GPX4 in BEAS-2B cells. **(G–I)** IHC images for GPX4 and 4-HNE expression in lung tissues. **(J)** Fe^2+^ expression in BEAS-2B cells. **(K)** TEM imaged showed ferroptosis of lung tissues. The results were represented by mean ± SD. ## *p* < 0.01, ** *p* < 0.01, &&*p* < 0.01 in comparison with control, LPS and LPS + UA groups, separately.

Ferroptosis results from lipid peroxidation and iron deposition, leading to mitochondrial contraction. The ferroptosis level in Lung tissue was evaluated by using the contents of 4-HNE, GPX4, SLC7A11, and Fe^2+^ in the LPS-mediated ALI model. UA treatment increased SLC7A11 and GPX4 in lung tissues and BEAS-2B cells compared to the LPS group (Figures 4A–D). The results of immunofluorescence evaluation of GPX4 and Fe^2+^ levels in BEAS-2B cells were consistent with WB results (Figures 4E, F, K). Additionally, SLC7A11 and GPX4 levels were increased in UA-treated BEAS-2B cells and mouse lung tissues (Supplementary Figures S4A–D). GPX4 expression significantly declined, while 4-HNE expression significantly increased in the LPS group, accompanied by distinct mitochondrial contraction (a typical morphological characteristics of ferroptosis) as shown by IHC staining and TEM analysis. Based on the above results, UA might reduce LPS-mediated ALI by suppressing ferroptosis (Figures 4G–J). Erastin is a putative activator for ferroptosis and can activate rapid, non-apoptotic, and oxidative cell death. As shown in Figure 4, Erastin was able to counteract UA’s inhibition of the ferroptosis in lung tissues and BEAS-2B cells. These findings suggested that UA ameliorated LPS-mediated injuries in BEAS-2B cells and ALI by inhibiting ferroptosis.

### UA Increased Keap1-Nrf2/HO-1 pathway in LPS-mediated BEAS-2B cells and ALI mice

**FIGURE 5:**
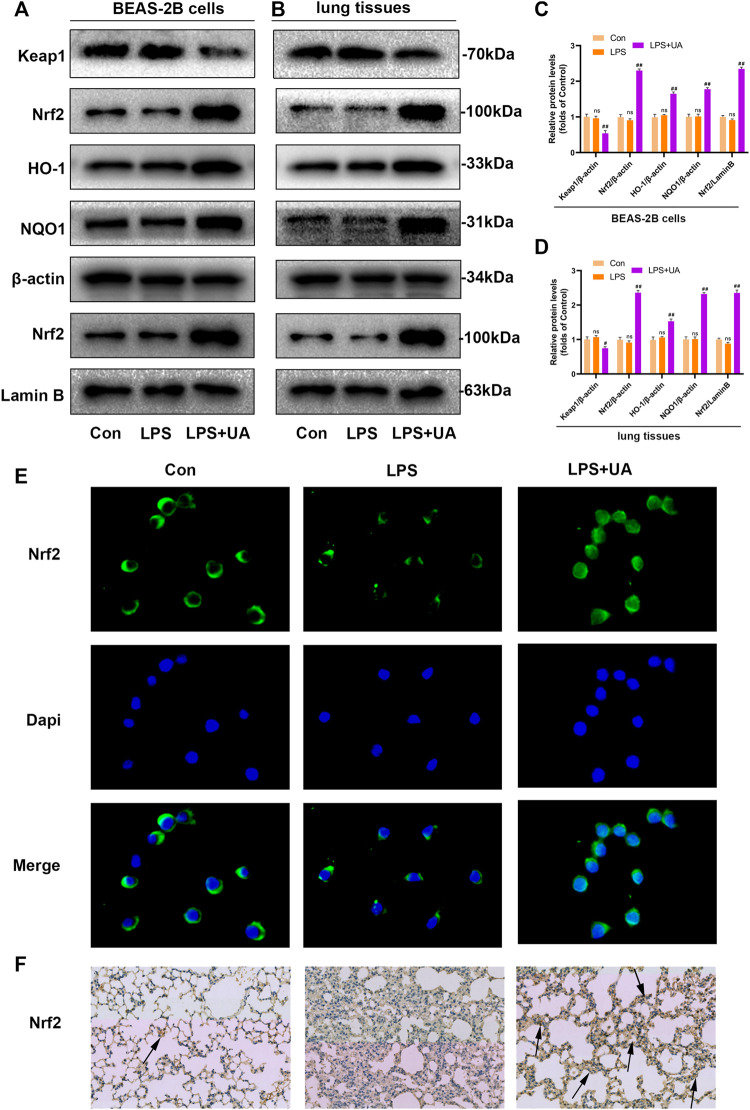
Urolithin A (UA) increased Nrf2 expression in LPS-mediated BEAS-2B cells and ALI models. **(A,C)** Quantification of Keap1, Nrf2, HO1, and NQO1 levels in BEAS-2B cells. **(B,D)** Quantification of Keap1, Nrf2, HO1, and NQO1 levels within lung tissues. **(E)** Representative immunofluorescence images showed Nrf2 in BEAS-2B cells. **(F)** Typical IHC images showed the Nrf2 in lung tissues. The results were represented by mean ± SD. ## *p* < 0.01 and ns in comparison to LPS and control groups, respectively.

Nrf2 has been identified as the activator of the antioxidant response element (ARE) and an important transcription factor (TF) that regulates the antioxidant stress response. To evaluate the effect of UA on LPS-induced injuries in BEAS-2B cells and ALI, WB, IF, and IHC assays were carried out to analyze Nrf2 expression and distribution. UA treatment enhanced the degradation of Keap1, and nuclear import and expression of Nrf2. Besides this, the downstream genes NQO1 and HO-1 also showed significant up-regulation, which indicated that UA’s protection on LPS-mediated ALI was possibly associated with increased Nrf2 expression (Figures 5–D). The IF and IHC results of Nrf2 level in BEAS-2B cells and mouse lung tissue are consistent with WB results (Figures 5E, F). In addition, consistent with the above results, the Keap1/Nrf2-HO-1 signaling pathway was activated in UA-treated BEAS-2B cells and mouse lung tissues (Supplementary Figures S4A–D). These findings suggested that UA Increased Keap1-Nrf2/HO-1 pathway in LPS-mediated BEAS-2B Cells and ALI Mice.

### Molecular docking interaction of UA with Nrf2-Keap1

**FIGURE 6:**
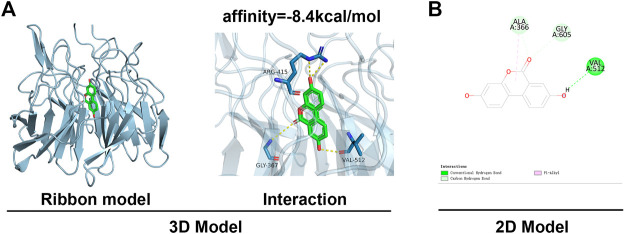
MD analysis showed that UA binds to Nrf2. **(A)** Ribbon and space-filling models of UA with Nrf2-Keap1 complex. **(B)** 2D binding models of UA with Nrf2-Keap1 complex.

This study was done by using *in silico* MD to analyze the effect of UA on the activation of Nrf2. The binding affinity was found to be −8.4 kcal mol^−1,^ and UA showed favorable embedding into the Keap1 structural domain that interacted with Nrf2 in Figure 6A, with local interaction images showing a hydrogen bond between UA and ALA366 residue was formed. Moreover, the 2D model clearly showed the formation of massive hydrophobic bonds between TF and diverse surrounding residues, namely, ALA 366, GLY 605, and VAL 512. The results indicated a strong affinity between UA and Keap1-Nrf2 complex protein.

### UA suppressed ferroptosis by activating Nrf2

**FIGURE 7:**
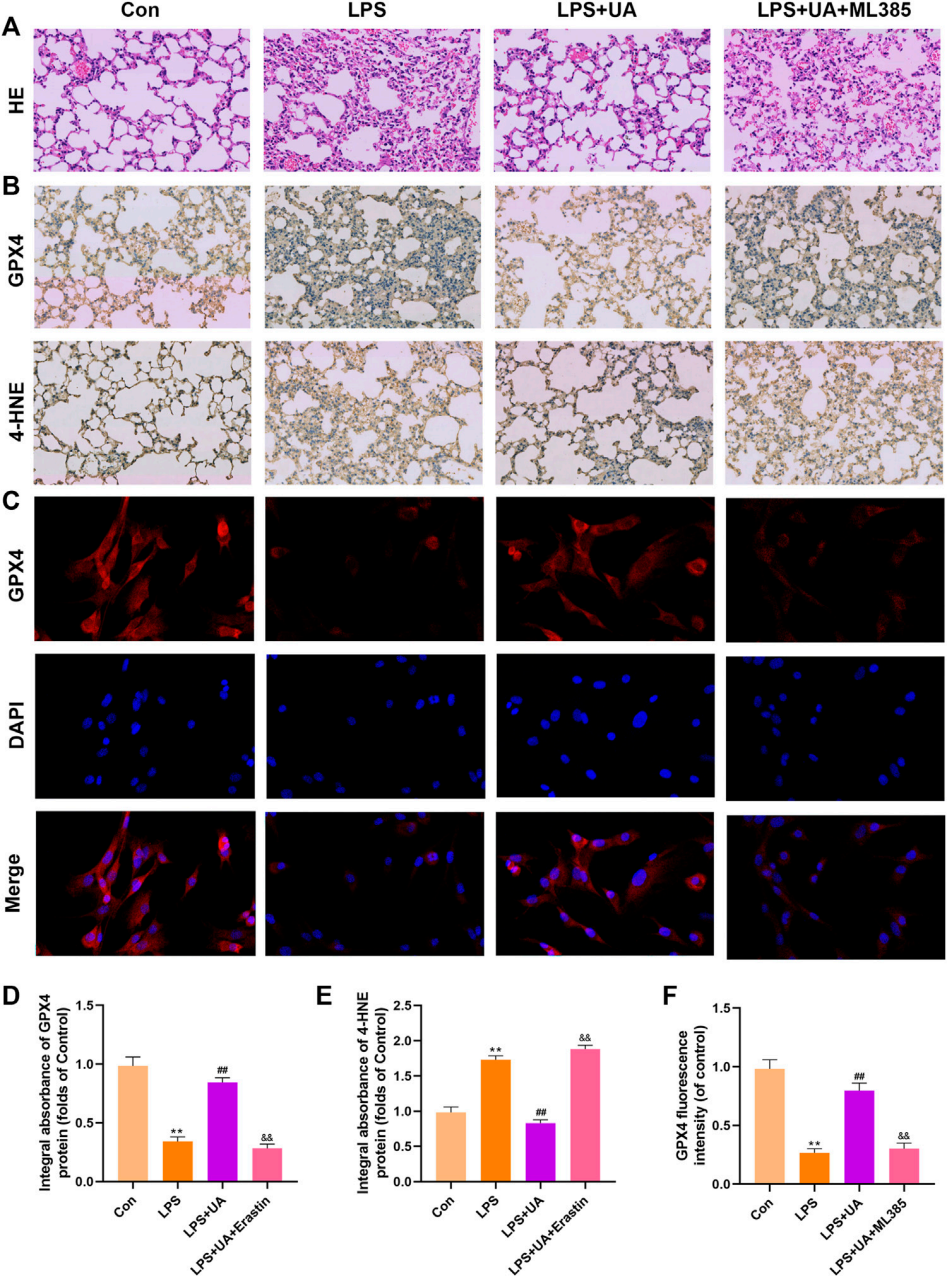
Urolithin A (UA) inhibited ferroptosis by activating Nrf2. **(A)** Typical images showed the H&E staining in lung tissue. **(B,D,E)** A typical IHC image showed the GPX4 and 4-HNE expression in lung tissues. **(C,F)** Typical IF images showed GPX4 expression in BEAS-2B cells. The results were represented by mean ± SD. ## *p* < 0.01, ** *p* < 0.01, &&*p* < 0.01 in comparison to control, LPS and LPS + UA groups, respectively.

To confirm how UA works to protect against ALI by activating Nrf2. Mice were pretreated with the Nrf2 inhibitor (ML385) at a dose of 30 mg/kg for 2 h, and BEAS-2B cells were treated with 5 µm ML385 for a period of 24 h, as described earlier, followed by UA treatment. Figure 7, 8 showed that ML385 significantly decreased UA’s ability to protect against inflammatory response, ferroptosis, and OS. Consequently, Nrf2 has a critical effect on the development of LPS-mediated ALI. In addition, Nrf2 activation by UA may help to decrease OS and inflammation, while suppressing ferroptosis in LPS-mediated BEAS-2B injury and ALI.

**FIGURE 8:**
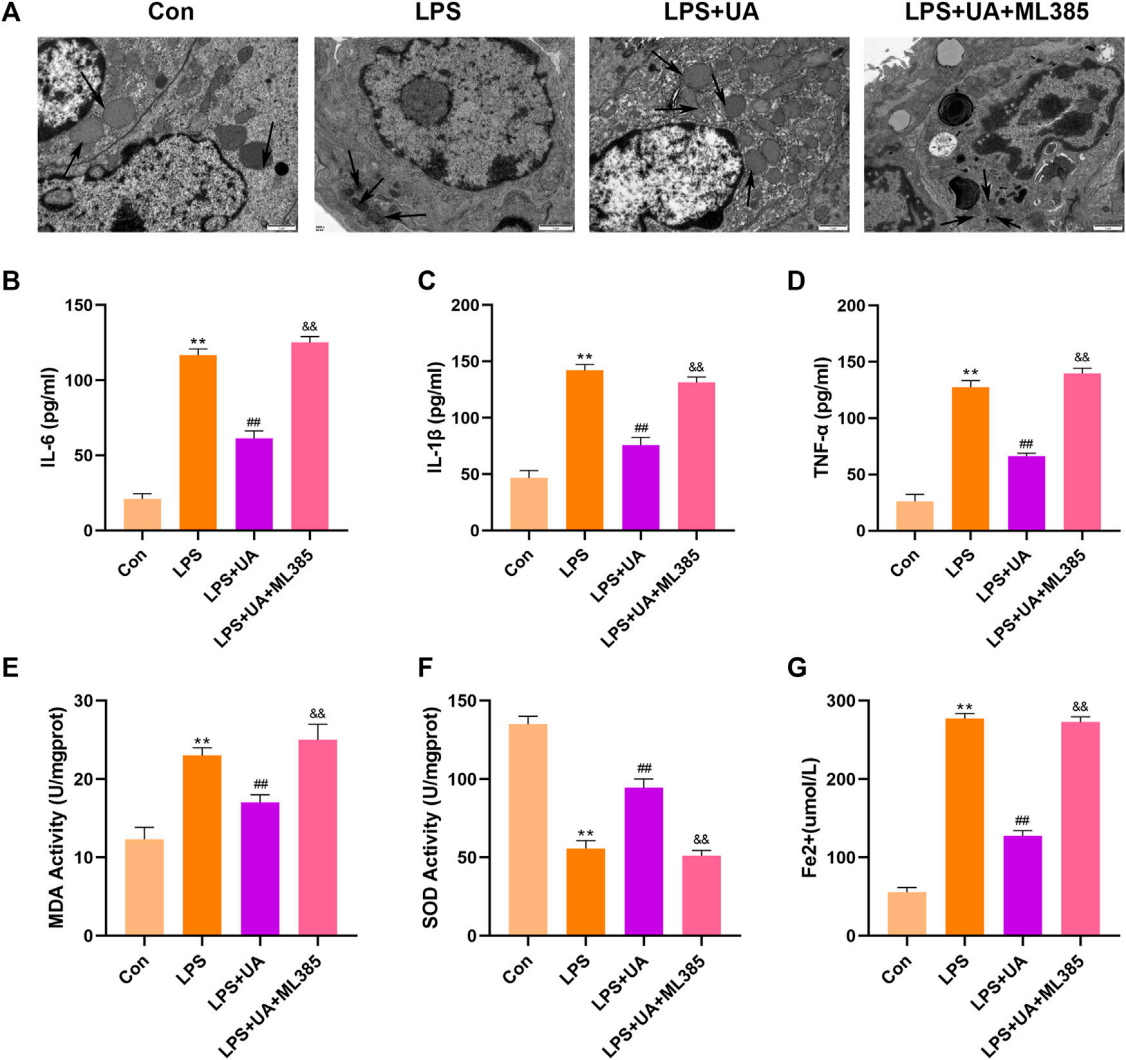
Urolithin A (UA) inhibited ferroptosis by activating Nrf2. **(A)** Typical TEM images showed ferroptosis in the lung tissues. **(B–D)** TNF-*α*, IL-1β, and IL-6 concentrations in the supernatants BEAS-2B cells. **(E,F)** SOD and MDA activities in lung tissues. **(G)** Fe^2+^ expression in BEAS-2B cells. The results were represented by mean ± SD. ## *p* < 0.01, ** *p* < 0.01, &&*p* < 0.01 in comparison to control, LPS and LPS + UA groups, respectively.

## Discussion

ALI/ARDS induces serious pulmonary diseases, pulmonary presentation of multiple organ dysfunction syndromes (MODS), as well as uncontrollable, self-amplified lung inflammation (Ferguson et al., 2012; Matthay et al., 2020). The major pathogenic mechanism of ALI/ARDS is uncontrolled lung or systemic inflammation (Janz and Ware, 2013). Among inpatients, ALI results in annual mortality of 4%, while that is 10% among intensive care unit (ICU) cases (Bellani et al., 2016). Despite the fact that numerous medicines have been investigated. There is currently no effective pharmaceutical treatment for ALI/ARDS that can significantly increase life quality and lower mortality (Ortiz-Diaz et al., 2013; Fan et al., 2018). In this regard, developing novel therapeutic agents to treat ALI/ARDS is necessary. Our results showed that UA efficiently promoted Keap1-Nrf2/HO-1 signaling pathways while suppressing ferroptosis, suggesting that it might be the potential anti-ALI therapeutic agent.

UA is an intestinal metabolite produced by ellagic acid-rich foods such as pomegranates, walnuts, and berries. It is known for various biological activities, such as anti-inflammatory and antioxidant properties (Espin et al., 2013; Wong et al., 2021). However, due to differences in gut microbiota composition between individuals, the amount of UA produced in bodies can vary significantly (Selma et al., 2017). Some populations do not even produce UA due to a lack of corresponding gut microbiota. Therefore, proper UA dosage is required in light of its safety and beneficial effects (Lin et al., 2020). Despite this, the role of UA in ALI is unknown. The current study analyzed the effect of UA on LPS-mediated ALI. Therefore, UA treatment significantly reduced the LPS-mediated pathological changes, edema, inflammation, and oxidative stress in the lungs.

The regulated form of necrosis known as ferroptosis is brought on by iron toxicity, plasma membrane damage, and lipid peroxidation (Berke et al., 2021). Ferroptosis is extensively suggested to promote ALI development (Dong et al., 2020; Li et al., 2020; Liu et al., 2020; Qiu et al., 2020). UA has been widely implicated in oxidative stress and lipid peroxidation (Casedas et al., 2020). In our study, we investigated the impact of ferroptosis on LPS-mediated ALI. As a result, ferroptosis developed in LPS-induced ALI, and UA significantly decreased LPS-induced ferroptosis. Erastin is an experimentally verified drug for inducing ferroptosis (Sehm et al., 2016; Roh et al., 2017). The ferroptosis-inducing small molecule erastin significantly decreased GSH expression in cells, thereby promoting ferroptosis, lipid peroxidation, and loss of protective GPX4 expression. Interestingly, UA reversed this effect, suggesting that it might be used as a treatment because it protects by inhibiting LPS-mediated ferroptosis.

This study also analyzed the mechanisms related to UA’s effect on LPS-mediated ALI. According to a recent study, UA inhibited hepatotoxicity caused by acetaminophen by increasing OS and activating Nrf2(Gao et al., 2022). It is now known that Nrf2 plays a key role in regulating antioxidant defense, which is also related to several cell death pathways (Kaminskyy and Zhivotovsky, 2014; Dodson et al., 2019). The most important transcriptional regulators in anti-ferroptotic pathways include Nrf2(Kerins and Ooi, 2018). It is also a crucial element in regulating ferroptosis and lipid peroxidation. Nrf2 is activated by electrophilic modification of KEAP1, which inhibits ferroptosis and lipid peroxidation and negatively regulates Nrf2 Normally, Nrf2 shows the cytoplasmic location, which can connect to Keap1 and maintain a low level of Nrf2 expression (Dinkova-Kostova et al., 2017). When stimulated externally, Nrf2 will be dissociated from Keap1, enters the nucleus, activates downstream molecule HO-1, increases the expression of SOD, CAT, and GSH, and defends against oxidative stress (Kensler et al., 2007; Niso-Santano et al., 2010; Gong and Yang, 2020; Zhao et al., 2021). An increasing body of evidence pointed out that numerous therapeutic agents inhibit ferroptosis by activating the Nrf2. For instance, obacunone boosted Nrf2-mediated antioxidant responses to reduce ferroptosis in LPS-mediated ALI increase (J. Li et al., 2022). Leonurine also reduced ferroptosis in the cisplatin-mediated AKI by activating the Nrf2 pathway (Hu et al., 2022). By stimulating Nrf2, TBHQ reduced ferroptosis’ effect on 5-fluorouracil-mediated intestinal mucositis and damage to intestinal epithelial cells (Deng et al., 2021). In this study, UA treatment led to Keap1-Nrf2/HO-1 pathways activation in LPS-induced acute lung injury ferroptosis, while inhibiting the Keap1-Nrf2/HO-1 pathways significantly abolished trehalose’s function in suppressing ferroptosis. As a result, UA reduced the ferroptosis and inflammation in LPS-mediated ALI by activating Keap1-Nrf2/HO-1 pathways. UA, the novel agonist for Nrf2, inhibited OS resulting from LPS-mediated ALI, which may become the candidate therapeutic target for ALI.

However, this study has some limitations. In addition to BEAS-2B cells, *in vivo* protective effects of UA on endothelial cells and macrophages may have other cytoprotective modalities. Finally, the current study is limited to animal models and *in vitro* studies, and there is no clinical evidence. More comprehensive and in-depth scientific studies will be required to further investigate the relationship between UA and iron death in ALI and provide a theoretical foundation for clinical work.

## Conclusion

**FIGURE 9:**
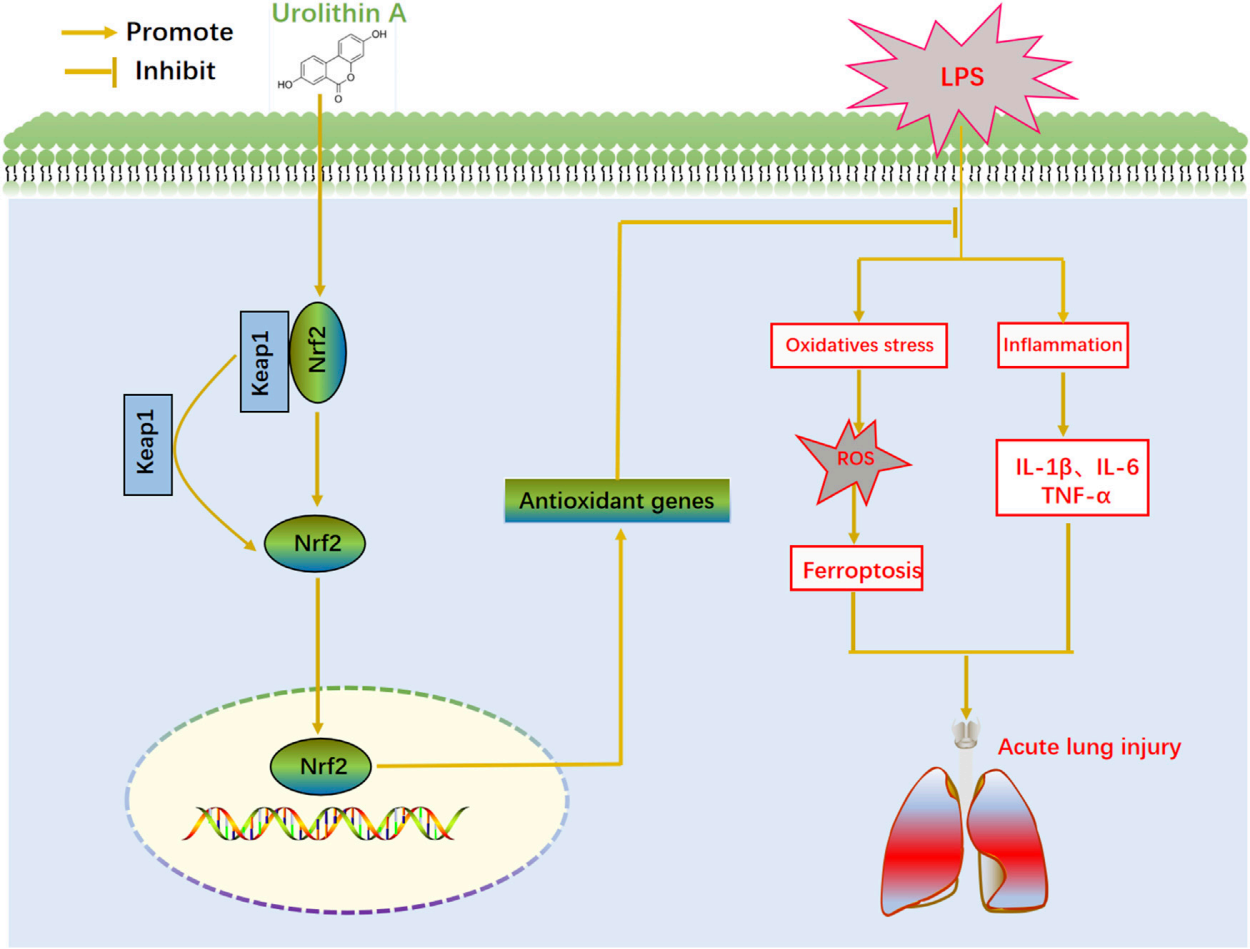
Schematic illustration of effect of UA in LPS-induced ALL.

In summary, UA significantly reduced histopathological changes, W/D weight ratio, inflammatory cell infiltration, and protected from LPS-mediated mouse ALI. UA increased the level of antioxidants in lung tissues while reducing LPS-mediated ferroptosis by activating Keap1-Nrf2/HO-1 pathway; thus, protecting from LPS-mediated ALI (Figure 9). This study suggested that UA can be potentially used to prevent ALI, offering exciting prospective applications.

## Data Availability

The original contributions presented in the study are included in the article/Supplementary Material, further inquiries can be directed to the corresponding author.
