# Supplementary figures and images for: Urolithin A (UA) attenuates ferroptosis in LPS-induced acute lung injury in mice by upregulating Keap1-Nrf2/HO-1 signaling pathway
Source: Front Pharmacol. 2023 Mar 9;14:1067402. doi: 10.3389/fphar.2023.1067402 (PMC10034769; doi:10.3389/fphar.2023.1067402)

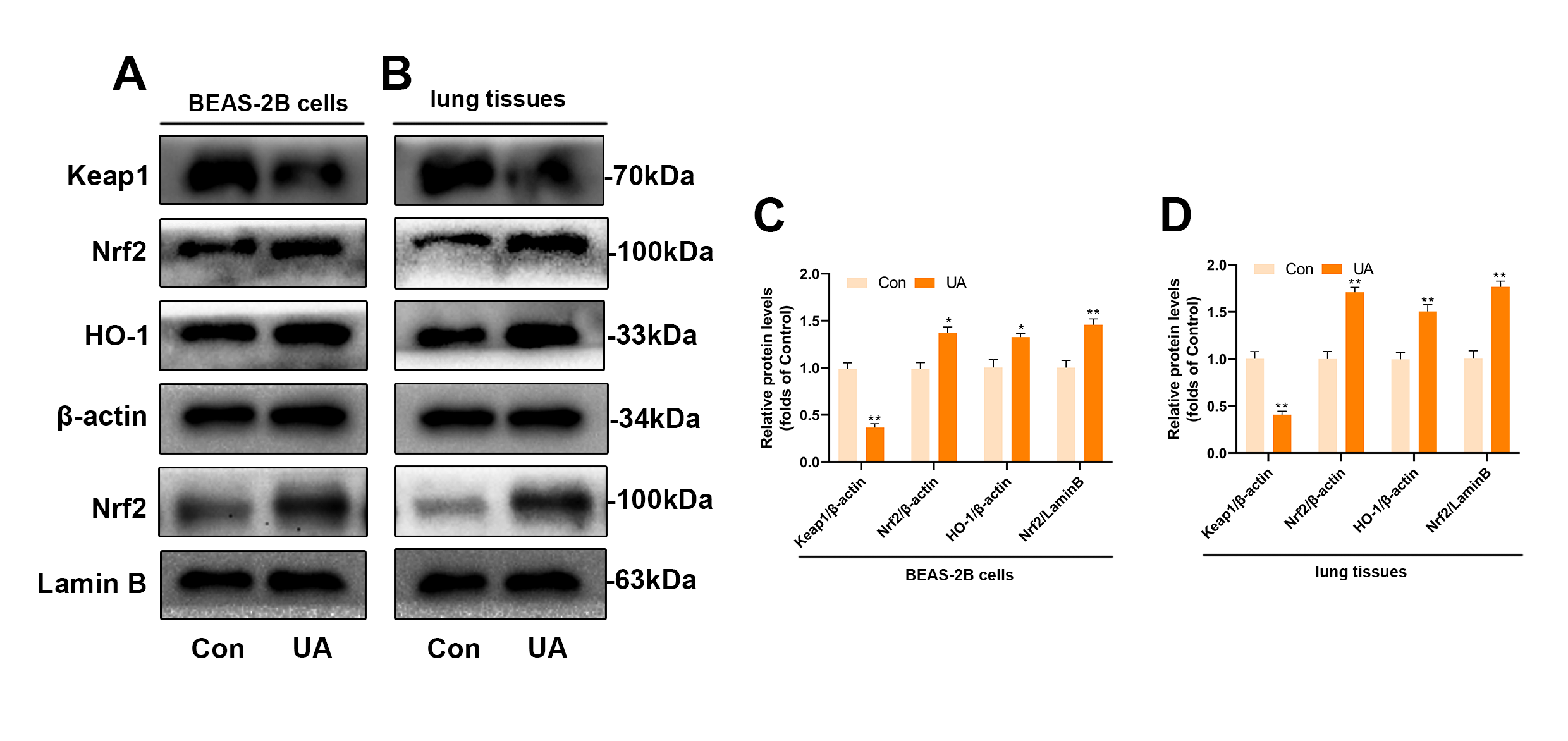

Supplement: Supplementary file 1 [file Image2.tif]

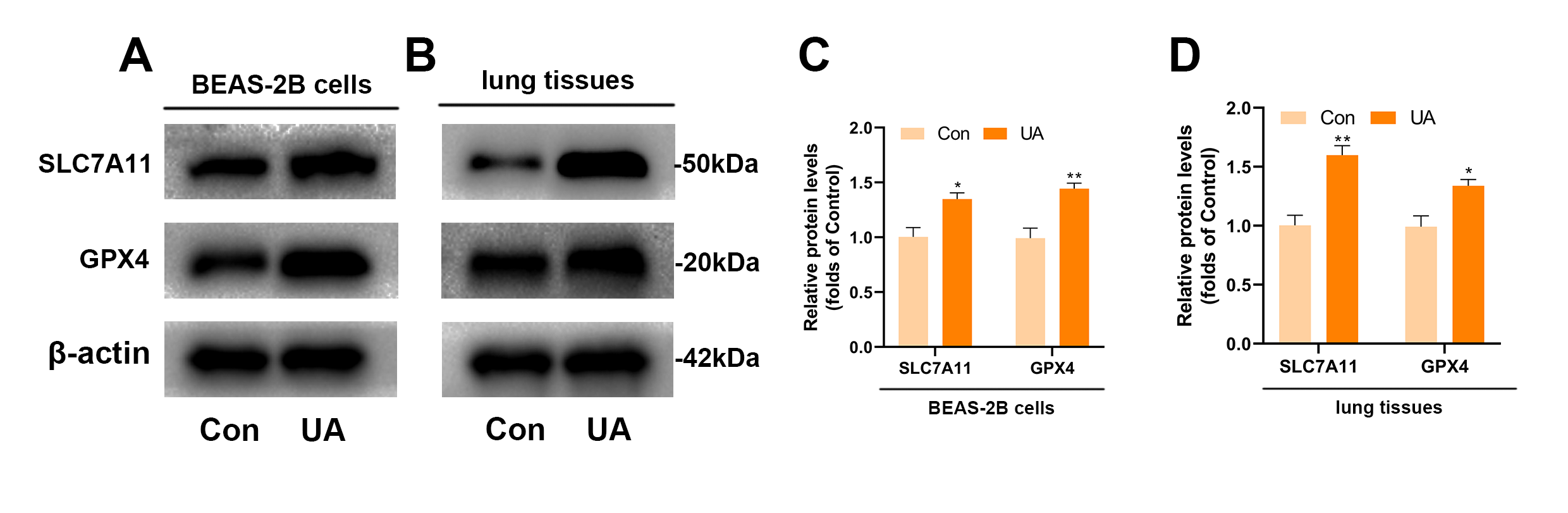

Supplement: Supplementary file 2 [file Image1.tif]
